# Excessive Hemorrhage after Extraction of Teeth—A Case

**Published:** 1890-02

**Authors:** C. H. M. Neall, T. D. Dunn

**Affiliations:** Philadelphia; Weschester, Pa.


ARTICLE VI.

### EXCESSIVE HEMORRHAGE AFTER EXTRACTION OF TEETH—A CASE.

BY C. H. M. NEALL, M. D., D. D. S., PHILADELPHIA, WITH REMARKS BY T. D. DUNN, M. D., WESCHESTER, PA.

Some time ago an elderly woman called at my dental office to have some teeth extracted. She enjoyed excellent health, and is the mother of two children. I gave her an anæsthetic—nitrous oxide gas—and extracted on the right side of the upper jaw the second bicuspid and the first molar, and on the left side the first and second bicuspids, and the first, second, and third molars.

Before leaving my office, the hemorrhage ceased. About two hours later the patient returned with copious hemorrhage from the right side of the upper jaw, but no hemorrhage from the opposite side. I cleaned out the cavities separately, and applied Monsell's solution on cotton.

The blood pressure was so strong as to wash the compress out, completely, of the cavities. Finally, by strong compression by means of a roller bandage, I checked it to a certain extent. The hemorrhage then came out of the ear. The patient was very much exsanguinated from the loss of blood. I gave internally gallic acid with ergot. After I had checked the hemorrhage, I applied the actual cautery to the alveolar cavities. It was fully one hour from the time the patient came in until she departed for her home. I was told by her physician that after she arrived home—which was about twenty miles from the city—the hemorrhage returned, and that he had the same trouble that I had had. I cannot understand why the hemorrhage was only from the right side. I pulled out more teeth on the left and larger ones. I have heard that this woman would bleed for hours if she cut any portion of her body, or even scratched her hand. This is said to occur only when she is cut upon the right side of the body. If she happens to cut herself on the left side, she will bleed only a short time.

REMARKS BY DR. T. D. DUNN.

Unfortunately, in Dr. Neall's interesting notes there is no mention of a family disposition or tendency to bleed, without which there must be some uncertainty whether or not the patient is a true subject of the congenital and hereditary diseases, hemophilia, which is the most hereditary of all diseases. The most remarkable family of bleeders on record lived in the village of Tenna, in the canton of Graubundten, as described by Anton Hœssli, who has traced the disease in his family to the year 1640, or for nearly 300 years. I think the fact of the bleeding being confined to the right side of the body in Dr. Neall's case is a mere coincidence I can find no record of unilateral hemorrhage in this disease, and it would not be in accordance with our knowledge of its pathology. Probably, as has been pointed out, "two circumstances combine in hemophilia—a congenital frangibility of the vessels, and a defect in the coagulability of the blood; but whereon these depend we are as yet entirely ignorant."

The tendency to bleed after the extraction of the teeth, as after all minor operations, is exceedingly common in hemophilia Hemophilists seem predisposed to rheumatic toothache, and frequently apply to dentists who are unaware of the bleeding tendency, instead of their medical adviser. They should, therefore, be especially warned of this danger by the family physician.

The treatment of Dr. Neall's case was very judicious. In several recorded cases, after the introduction of cotton into the alveolus, a cork has been trimmed to fit the cavity and by closing the jaw, held sufficiently firm, to control bleeding, the pressure being gradually withdrawn as the hemorrhage ceases. Ranger, in St. Thomas's Hospital Reports, vol. vi, p. 121, advises that an impression of the jaw should be taken with plaster of Paris mixed with salt and warm water, the jaw to be held in position by a compress and bandage. Before taking the impression the mouth should be thoroughly cleansed of blood.

The quantity of blood lost after extraction of teeth has in some cases been enormous. Krimes reports a case in which four and a half pounds of blood were lost in twenty-four hours, and Schaefer's case lost four pounds daily for several days after an extraction. Dr. R. Coates relates a case in the *North American Medical and Surgical Journal*, vol vi, page 45, 1828, in which three gallons (24 lbs.) were lost in eleven days. Several bleeder descendants of Dr. Coates' patient have come under my observation and are reported in the *American Journal of the Medical Sciences*, January, 1883. The marked tolerance of bleeders for great losses of blood and the rapidity with which they recover after them are even more remarkable than the severity of the hemmorrhages.—*Med. and Surg. Rep.*

